# Supplementary material for: Multi‐dataset identification of innovative feature genes and molecular mechanisms in keratoconus
Source: J Cell Mol Med. 2024 Sep 19;28(18):e70079. doi: 10.1111/jcmm.70079 (PMC11412914; doi:10.1111/jcmm.70079)
Supplement: Supplementary file 2 — Material S2. [file JCMM-28-e70079-s002.zip › 7.GSEA analysis/6.SNORA74B/KEGG_MAPK_SIGNALING_PATHWAY.html]

Details for gene set KEGG\_MAPK\_SIGNALING\_PATHWAY[GSEA]

|  || Dataset | SNORA74B.group.cls#h\_versus\_l |
| Phenotype | group.cls#h\_versus\_l |
| Upregulated in class | l |
| GeneSet | KEGG\_MAPK\_SIGNALING\_PATHWAY |
| Enrichment Score (ES) | -0.35900083 |
| Normalized Enrichment Score (NES) | -1.4696045 |
| Nominal p-value | 0.026768642 |
| FDR q-value | 0.30348969 |
| FWER p-Value | 0.824 |
Table: GSEA Results Summary

  

Fig 1: Enrichment plot: KEGG\_MAPK\_SIGNALING\_PATHWAY      
 Profile of the Running ES Score & Positions of GeneSet Members on the Rank Ordered List

  

| SYMBOL | TITLE | RANK IN GENE LIST | RANK METRIC SCORE | RUNNING ES | CORE ENRICHMENT || 1 | CACNB1 | na | 316 | 0.683 | 0.0073 | No |
| 2 | NFATC2 | na | 1406 | 0.425 | -0.0140 | No |
| 3 | CACNA1I | na | 1457 | 0.420 | -0.0053 | No |
| 4 | RAC3 | na | 1795 | 0.390 | -0.0056 | No |
| 5 | FGF18 | na | 1820 | 0.388 | 0.0030 | No |
| 6 | PPP3R2 | na | 2054 | 0.366 | 0.0051 | No |
| 7 | ELK1 | na | 2088 | 0.363 | 0.0129 | No |
| 8 | PRKCG | na | 2539 | 0.330 | 0.0079 | No |
| 9 | FGF8 | na | 2974 | 0.302 | 0.0026 | No |
| 10 | FGF5 | na | 3327 | 0.285 | -0.0008 | No |
| 11 | RASGRF2 | na | 3343 | 0.284 | 0.0057 | No |
| 12 | CACNG8 | na | 3573 | 0.275 | 0.0057 | No |
| 13 | FGF17 | na | 3644 | 0.272 | 0.0102 | No |
| 14 | CACNG5 | na | 3979 | 0.261 | 0.0068 | No |
| 15 | CACNG7 | na | 4118 | 0.256 | 0.0090 | No |
| 16 | PDGFRA | na | 4146 | 0.255 | 0.0144 | No |
| 17 | CHP2 | na | 4857 | 0.230 | -0.0007 | No |
| 18 | PLA2G4E | na | 5383 | 0.212 | -0.0108 | No |
| 19 | FGF16 | na | 5815 | 0.198 | -0.0185 | No |
| 20 | DUSP9 | na | 5902 | 0.196 | -0.0162 | No |
| 21 | SOS1 | na | 5971 | 0.193 | -0.0136 | No |
| 22 | FLNB | na | 6092 | 0.190 | -0.0125 | No |
| 23 | CACNA1F | na | 6293 | 0.183 | -0.0138 | No |
| 24 | RPS6KA2 | na | 6719 | 0.170 | -0.0221 | No |
| 25 | RASGRP2 | na | 7019 | 0.162 | -0.0268 | No |
| 26 | TAOK2 | na | 7291 | 0.154 | -0.0310 | No |
| 27 | FGF3 | na | 7481 | 0.149 | -0.0329 | No |
| 28 | CACNA2D3 | na | 7638 | 0.145 | -0.0339 | No |
| 29 | BRAF | na | 8089 | 0.134 | -0.0437 | No |
| 30 | MAPK11 | na | 8296 | 0.129 | -0.0466 | No |
| 31 | HSPA2 | na | 8508 | 0.124 | -0.0497 | No |
| 32 | HSPA8 | na | 8644 | 0.121 | -0.0507 | No |
| 33 | IKBKB | na | 8763 | 0.119 | -0.0512 | No |
| 34 | FGFR4 | na | 9201 | 0.109 | -0.0612 | No |
| 35 | RASA1 | na | 9229 | 0.109 | -0.0594 | No |
| 36 | ARRB1 | na | 9230 | 0.109 | -0.0568 | No |
| 37 | MAP3K6 | na | 9300 | 0.107 | -0.0562 | No |
| 38 | RASGRF1 | na | 9313 | 0.107 | -0.0540 | No |
| 39 | AKT2 | na | 9380 | 0.105 | -0.0533 | No |
| 40 | TGFBR2 | na | 9513 | 0.102 | -0.0547 | No |
| 41 | MAP3K4 | na | 9531 | 0.102 | -0.0527 | No |
| 42 | CACNG1 | na | 9619 | 0.100 | -0.0528 | No |
| 43 | FASLG | na | 9725 | 0.098 | -0.0535 | No |
| 44 | KRAS | na | 9766 | 0.097 | -0.0523 | No |
| 45 | NF1 | na | 9786 | 0.097 | -0.0505 | No |
| 46 | CASP3 | na | 9789 | 0.097 | -0.0482 | No |
| 47 | PDGFA | na | 9861 | 0.095 | -0.0480 | No |
| 48 | CACNB2 | na | 10102 | 0.091 | -0.0528 | No |
| 49 | PLA2G12B | na | 10125 | 0.090 | -0.0512 | No |
| 50 | CACNA1D | na | 10286 | 0.087 | -0.0538 | No |
| 51 | MECOM | na | 10361 | 0.086 | -0.0538 | No |
| 52 | ELK4 | na | 10367 | 0.086 | -0.0519 | No |
| 53 | RPS6KA5 | na | 10419 | 0.085 | -0.0514 | No |
| 54 | MAPK9 | na | 10427 | 0.085 | -0.0495 | No |
| 55 | NTRK1 | na | 10524 | 0.083 | -0.0503 | No |
| 56 | PLA2G10 | na | 10767 | 0.079 | -0.0554 | No |
| 57 | CACNA1C | na | 10871 | 0.077 | -0.0565 | No |
| 58 | PPM1B | na | 10904 | 0.077 | -0.0556 | No |
| 59 | FGF20 | na | 10959 | 0.076 | -0.0554 | No |
| 60 | MAPK13 | na | 11114 | 0.073 | -0.0581 | No |
| 61 | FGFR3 | na | 11176 | 0.072 | -0.0581 | No |
| 62 | TGFB2 | na | 11803 | 0.063 | -0.0747 | No |
| 63 | MAPK12 | na | 11855 | 0.062 | -0.0747 | No |
| 64 | FGF9 | na | 12036 | 0.059 | -0.0785 | No |
| 65 | RPS6KA4 | na | 12098 | 0.059 | -0.0789 | No |
| 66 | MKNK1 | na | 12441 | 0.054 | -0.0875 | No |
| 67 | MKNK2 | na | 12656 | 0.051 | -0.0925 | No |
| 68 | PTPN5 | na | 12716 | 0.050 | -0.0930 | No |
| 69 | MAPK14 | na | 12827 | 0.049 | -0.0950 | No |
| 70 | TAOK1 | na | 13538 | 0.041 | -0.1146 | No |
| 71 | SOS2 | na | 13685 | 0.039 | -0.1179 | No |
| 72 | FGF2 | na | 13739 | 0.039 | -0.1184 | No |
| 73 | FGF11 | na | 13944 | 0.036 | -0.1235 | No |
| 74 | JMJD7-PLA2G4B | na | 13953 | 0.036 | -0.1228 | No |
| 75 | DUSP3 | na | 13991 | 0.036 | -0.1230 | No |
| 76 | MAPKAPK3 | na | 14265 | 0.033 | -0.1302 | No |
| 77 | RPS6KA3 | na | 14434 | 0.031 | -0.1343 | No |
| 78 | MAPK8IP3 | na | 14528 | 0.030 | -0.1363 | No |
| 79 | PDGFRB | na | 14565 | 0.029 | -0.1366 | No |
| 80 | MAP3K3 | na | 14575 | 0.029 | -0.1362 | No |
| 81 | FGF22 | na | 14606 | 0.029 | -0.1363 | No |
| 82 | MAP2K2 | na | 14904 | 0.025 | -0.1443 | No |
| 83 | MAPK10 | na | 14996 | 0.024 | -0.1464 | No |
| 84 | FGF13 | na | 15000 | 0.024 | -0.1459 | No |
| 85 | TRAF2 | na | 15165 | 0.023 | -0.1501 | No |
| 86 | CACNG2 | na | 15359 | 0.021 | -0.1552 | No |
| 87 | MAX | na | 15362 | 0.021 | -0.1547 | No |
| 88 | DAXX | na | 15562 | 0.019 | -0.1601 | No |
| 89 | TAOK3 | na | 15705 | 0.018 | -0.1637 | No |
| 90 | MAP3K11 | na | 15803 | 0.017 | -0.1662 | No |
| 91 | ARRB2 | na | 15989 | 0.015 | -0.1712 | No |
| 92 | MAP2K4 | na | 16268 | 0.012 | -0.1790 | No |
| 93 | PLA2G12A | na | 16297 | 0.012 | -0.1795 | No |
| 94 | CD14 | na | 16315 | 0.011 | -0.1797 | No |
| 95 | PLA2G6 | na | 16344 | 0.011 | -0.1803 | No |
| 96 | CHUK | na | 16402 | 0.010 | -0.1817 | No |
| 97 | HSPA1A | na | 16403 | 0.010 | -0.1814 | No |
| 98 | MAPK7 | na | 16513 | 0.009 | -0.1843 | No |
| 99 | MAPK3 | na | 16753 | 0.007 | -0.1911 | No |
| 100 | MOS | na | 16949 | 0.005 | -0.1966 | No |
| 101 | PLA2G2F | na | 17001 | 0.005 | -0.1980 | No |
| 102 | MAP2K7 | na | 17007 | 0.005 | -0.1980 | No |
| 103 | HSPA1B | na | 17184 | 0.003 | -0.2031 | No |
| 104 | FGF10 | na | 17212 | 0.003 | -0.2038 | No |
| 105 | FGF19 | na | 17258 | 0.002 | -0.2050 | No |
| 106 | EGF | na | 17351 | 0.001 | -0.2077 | No |
| 107 | RAPGEF2 | na | 17524 | -0.001 | -0.2127 | No |
| 108 | STK3 | na | 17741 | -0.003 | -0.2189 | No |
| 109 | RAP1A | na | 17815 | -0.004 | -0.2209 | No |
| 110 | AKT1 | na | 18366 | -0.010 | -0.2366 | No |
| 111 | RELB | na | 18391 | -0.011 | -0.2370 | No |
| 112 | RAP1B | na | 18593 | -0.013 | -0.2426 | No |
| 113 | HSPB1 | na | 18600 | -0.013 | -0.2424 | No |
| 114 | PLA2G2E | na | 18608 | -0.013 | -0.2423 | No |
| 115 | ATF4 | na | 18691 | -0.014 | -0.2444 | No |
| 116 | CDC42 | na | 18770 | -0.015 | -0.2463 | No |
| 117 | STK4 | na | 18825 | -0.015 | -0.2475 | No |
| 118 | RAC2 | na | 18892 | -0.016 | -0.2490 | No |
| 119 | NLK | na | 18915 | -0.016 | -0.2493 | No |
| 120 | MAP3K1 | na | 18950 | -0.016 | -0.2498 | No |
| 121 | MAP2K3 | na | 18965 | -0.017 | -0.2498 | No |
| 122 | CACNA1H | na | 19184 | -0.019 | -0.2557 | No |
| 123 | FGF14 | na | 19449 | -0.022 | -0.2628 | No |
| 124 | PLA2G2D | na | 19469 | -0.023 | -0.2628 | No |
| 125 | PPP3R1 | na | 19504 | -0.023 | -0.2633 | No |
| 126 | TP53 | na | 19572 | -0.024 | -0.2646 | No |
| 127 | EGFR | na | 19596 | -0.024 | -0.2647 | No |
| 128 | HRAS | na | 19699 | -0.025 | -0.2671 | No |
| 129 | RPS6KA6 | na | 19762 | -0.026 | -0.2682 | No |
| 130 | MAP2K5 | na | 19766 | -0.026 | -0.2677 | No |
| 131 | GADD45G | na | 19816 | -0.026 | -0.2685 | No |
| 132 | FOS | na | 19844 | -0.027 | -0.2686 | No |
| 133 | RAC1 | na | 19881 | -0.027 | -0.2690 | No |
| 134 | MAPK8 | na | 20414 | -0.033 | -0.2836 | No |
| 135 | FGF12 | na | 20421 | -0.033 | -0.2830 | No |
| 136 | FGF7 | na | 20472 | -0.034 | -0.2837 | No |
| 137 | TAB1 | na | 20492 | -0.034 | -0.2834 | No |
| 138 | MAPK1 | na | 20606 | -0.035 | -0.2858 | No |
| 139 | IKBKG | na | 20820 | -0.038 | -0.2911 | No |
| 140 | CACNA1B | na | 21005 | -0.040 | -0.2955 | No |
| 141 | PTPRR | na | 21062 | -0.041 | -0.2961 | No |
| 142 | RAF1 | na | 21074 | -0.041 | -0.2954 | No |
| 143 | PRKX | na | 21076 | -0.041 | -0.2945 | No |
| 144 | MAPK8IP1 | na | 21281 | -0.043 | -0.2993 | No |
| 145 | CHP1 | na | 21507 | -0.046 | -0.3048 | No |
| 146 | RASGRP4 | na | 21904 | -0.051 | -0.3150 | No |
| 147 | PPP3CB | na | 21942 | -0.051 | -0.3149 | No |
| 148 | TAB2 | na | 22232 | -0.054 | -0.3219 | No |
| 149 | PRKCA | na | 22239 | -0.055 | -0.3208 | No |
| 150 | TNFRSF1A | na | 22241 | -0.055 | -0.3195 | No |
| 151 | ECSIT | na | 22429 | -0.057 | -0.3235 | No |
| 152 | TRAF6 | na | 22560 | -0.059 | -0.3259 | No |
| 153 | PRKACA | na | 22659 | -0.060 | -0.3273 | No |
| 154 | MAPK8IP2 | na | 22965 | -0.065 | -0.3345 | No |
| 155 | PPP3CA | na | 23024 | -0.066 | -0.3346 | No |
| 156 | MAP3K2 | na | 23080 | -0.067 | -0.3346 | No |
| 157 | MAPKAPK5 | na | 23154 | -0.068 | -0.3351 | No |
| 158 | MAP3K7 | na | 23787 | -0.078 | -0.3516 | No |
| 159 | GNG12 | na | 23995 | -0.081 | -0.3556 | No |
| 160 | HSPA1L | na | 24075 | -0.082 | -0.3559 | No |
| 161 | PLA2G4A | na | 24085 | -0.082 | -0.3542 | No |
| 162 | PPM1A | na | 24211 | -0.084 | -0.3558 | No |
| 163 | FGFR2 | na | 24244 | -0.085 | -0.3547 | No |
| 164 | MAP3K13 | na | 24275 | -0.085 | -0.3535 | No |
| 165 | DUSP16 | na | 24451 | -0.088 | -0.3565 | No |
| 166 | LAMTOR3 | na | 24530 | -0.090 | -0.3566 | Yes |
| 167 | RRAS | na | 24557 | -0.090 | -0.3551 | Yes |
| 168 | GNA12 | na | 24641 | -0.092 | -0.3553 | Yes |
| 169 | NR4A1 | na | 24651 | -0.092 | -0.3533 | Yes |
| 170 | FGF6 | na | 24652 | -0.092 | -0.3511 | Yes |
| 171 | DUSP6 | na | 24740 | -0.094 | -0.3514 | Yes |
| 172 | PPP5C | na | 24950 | -0.098 | -0.3551 | Yes |
| 173 | GRB2 | na | 24997 | -0.098 | -0.3540 | Yes |
| 174 | MAP4K2 | na | 25021 | -0.099 | -0.3523 | Yes |
| 175 | MAPKAPK2 | na | 25117 | -0.100 | -0.3526 | Yes |
| 176 | FGF1 | na | 25243 | -0.103 | -0.3538 | Yes |
| 177 | JUN | na | 25317 | -0.104 | -0.3534 | Yes |
| 178 | MAP4K1 | na | 25511 | -0.107 | -0.3564 | Yes |
| 179 | MYC | na | 25534 | -0.108 | -0.3544 | Yes |
| 180 | DUSP14 | na | 25658 | -0.110 | -0.3553 | Yes |
| 181 | NGF | na | 25719 | -0.111 | -0.3544 | Yes |
| 182 | RRAS2 | na | 25730 | -0.112 | -0.3520 | Yes |
| 183 | PAK2 | na | 25754 | -0.112 | -0.3499 | Yes |
| 184 | RASA2 | na | 25788 | -0.113 | -0.3482 | Yes |
| 185 | DUSP8 | na | 25833 | -0.113 | -0.3467 | Yes |
| 186 | CACNA1A | na | 25928 | -0.115 | -0.3467 | Yes |
| 187 | JUND | na | 26217 | -0.121 | -0.3521 | Yes |
| 188 | IL1A | na | 26246 | -0.122 | -0.3500 | Yes |
| 189 | MAP4K4 | na | 26260 | -0.122 | -0.3474 | Yes |
| 190 | NTF4 | na | 26519 | -0.128 | -0.3518 | Yes |
| 191 | NFKB1 | na | 26534 | -0.128 | -0.3491 | Yes |
| 192 | DUSP1 | na | 26539 | -0.129 | -0.3461 | Yes |
| 193 | PLA2G2C | na | 26678 | -0.132 | -0.3469 | Yes |
| 194 | CRK | na | 26740 | -0.134 | -0.3454 | Yes |
| 195 | GADD45A | na | 26779 | -0.134 | -0.3433 | Yes |
| 196 | FGF21 | na | 27099 | -0.142 | -0.3491 | Yes |
| 197 | TGFBR1 | na | 27137 | -0.142 | -0.3468 | Yes |
| 198 | PLA2G3 | na | 27299 | -0.147 | -0.3479 | Yes |
| 199 | MAP3K5 | na | 27483 | -0.151 | -0.3496 | Yes |
| 200 | CDC25B | na | 27564 | -0.153 | -0.3482 | Yes |
| 201 | STMN1 | na | 27672 | -0.156 | -0.3475 | Yes |
| 202 | RPS6KA1 | na | 27808 | -0.160 | -0.3476 | Yes |
| 203 | NFATC4 | na | 27880 | -0.162 | -0.3457 | Yes |
| 204 | FAS | na | 27948 | -0.163 | -0.3437 | Yes |
| 205 | NTF3 | na | 27963 | -0.164 | -0.3402 | Yes |
| 206 | CACNB3 | na | 27964 | -0.164 | -0.3362 | Yes |
| 207 | NRAS | na | 27969 | -0.164 | -0.3324 | Yes |
| 208 | ATF2 | na | 28075 | -0.167 | -0.3314 | Yes |
| 209 | NFKB2 | na | 28383 | -0.176 | -0.3361 | Yes |
| 210 | DUSP7 | na | 28467 | -0.178 | -0.3341 | Yes |
| 211 | PAK1 | na | 28478 | -0.179 | -0.3301 | Yes |
| 212 | PTPN7 | na | 28696 | -0.185 | -0.3319 | Yes |
| 213 | PPP3CC | na | 28713 | -0.186 | -0.3279 | Yes |
| 214 | FLNC | na | 28751 | -0.188 | -0.3244 | Yes |
| 215 | GADD45B | na | 28844 | -0.190 | -0.3225 | Yes |
| 216 | BDNF | na | 28985 | -0.194 | -0.3219 | Yes |
| 217 | MAP4K3 | na | 29195 | -0.200 | -0.3231 | Yes |
| 218 | CRKL | na | 29271 | -0.203 | -0.3204 | Yes |
| 219 | CACNG6 | na | 29429 | -0.207 | -0.3199 | Yes |
| 220 | RELA | na | 29651 | -0.214 | -0.3212 | Yes |
| 221 | DUSP4 | na | 29872 | -0.221 | -0.3222 | Yes |
| 222 | PDGFB | na | 30041 | -0.227 | -0.3216 | Yes |
| 223 | FLNA | na | 30062 | -0.228 | -0.3167 | Yes |
| 224 | TGFB1 | na | 30144 | -0.231 | -0.3135 | Yes |
| 225 | FGF23 | na | 30156 | -0.231 | -0.3082 | Yes |
| 226 | MAP3K8 | na | 30218 | -0.234 | -0.3043 | Yes |
| 227 | IL1B | na | 30302 | -0.237 | -0.3010 | Yes |
| 228 | PLA2G5 | na | 30479 | -0.245 | -0.3002 | Yes |
| 229 | MAP2K1 | na | 31129 | -0.275 | -0.3124 | Yes |
| 230 | MAP3K14 | na | 31321 | -0.285 | -0.3110 | Yes |
| 231 | HSPA6 | na | 31406 | -0.289 | -0.3065 | Yes |
| 232 | TGFB3 | na | 31437 | -0.291 | -0.3004 | Yes |
| 233 | CACNA1G | na | 31581 | -0.300 | -0.2973 | Yes |
| 234 | MAPT | na | 31663 | -0.305 | -0.2923 | Yes |
| 235 | CACNG4 | na | 31720 | -0.308 | -0.2865 | Yes |
| 236 | TNF | na | 31738 | -0.308 | -0.2795 | Yes |
| 237 | MAP2K6 | na | 31770 | -0.311 | -0.2729 | Yes |
| 238 | DUSP2 | na | 32257 | -0.338 | -0.2788 | Yes |
| 239 | PRKACB | na | 32349 | -0.344 | -0.2732 | Yes |
| 240 | DDIT3 | na | 32384 | -0.346 | -0.2658 | Yes |
| 241 | PRKACG | na | 32698 | -0.368 | -0.2660 | Yes |
| 242 | SRF | na | 32701 | -0.368 | -0.2571 | Yes |
| 243 | MAP3K12 | na | 32737 | -0.371 | -0.2492 | Yes |
| 244 | CACNA2D4 | na | 32845 | -0.380 | -0.2431 | Yes |
| 245 | IL1R1 | na | 32935 | -0.388 | -0.2363 | Yes |
| 246 | DUSP5 | na | 33057 | -0.400 | -0.2302 | Yes |
| 247 | CACNA1S | na | 33081 | -0.402 | -0.2211 | Yes |
| 248 | NTRK2 | na | 33295 | -0.423 | -0.2171 | Yes |
| 249 | RASGRP1 | na | 33606 | -0.465 | -0.2148 | Yes |
| 250 | CACNG3 | na | 33713 | -0.479 | -0.2063 | Yes |
| 251 | CACNA2D2 | na | 33786 | -0.491 | -0.1965 | Yes |
| 252 | PRKCB | na | 33882 | -0.508 | -0.1870 | Yes |
| 253 | CACNB4 | na | 33958 | -0.525 | -0.1765 | Yes |
| 254 | PLA2G2A | na | 33973 | -0.527 | -0.1642 | Yes |
| 255 | IL1R2 | na | 34002 | -0.532 | -0.1521 | Yes |
| 256 | DUSP10 | na | 34154 | -0.569 | -0.1428 | Yes |
| 257 | PLA2G1B | na | 34211 | -0.585 | -0.1303 | Yes |
| 258 | MEF2C | na | 34263 | -0.599 | -0.1173 | Yes |
| 259 | CACNA1E | na | 34274 | -0.602 | -0.1030 | Yes |
| 260 | FGF4 | na | 34349 | -0.632 | -0.0899 | Yes |
| 261 | RASGRP3 | na | 34424 | -0.662 | -0.0761 | Yes |
| 262 | MRAS | na | 34571 | -0.753 | -0.0621 | Yes |
| 263 | AKT3 | na | 34657 | -0.880 | -0.0433 | Yes |
| 264 | CACNA2D1 | na | 34671 | -0.912 | -0.0216 | Yes |
| 265 | FGFR1 | na | 34687 | -0.963 | 0.0012 | Yes |
Table: GSEA details [plain text format]

  

Fig 2: KEGG\_MAPK\_SIGNALING\_PATHWAY      
 Blue-Pink O' Gram in the Space of the Analyzed GeneSet

  

Fig 3: KEGG\_MAPK\_SIGNALING\_PATHWAY: Random ES distribution      
 Gene set null distribution of ES for **KEGG\_MAPK\_SIGNALING\_PATHWAY**
